# Structural analysis and functional evaluation of the disordered ß–hexosyltransferase region from *Hamamotoa (Sporobolomyces) singularis*

**DOI:** 10.3389/fbioe.2023.1291245

**Published:** 2023-12-14

**Authors:** Suzanne F. Dagher, Asmita Vaishnav, Christopher B. Stanley, Flora Meilleur, Brian F. P. Edwards, José M. Bruno-Bárcena

**Affiliations:** ^1^ Department of Plant and Microbial Biology, North Carolina State University, Raleigh, NC, United States; ^2^ Department of Biochemistry, Microbiology and Immunology, Wayne State University, Detroit, MI, United States; ^3^ Computational Sciences and Engineering Division, Oak Ridge, TN, United States; ^4^ Neutron Sciences Directorate, Oak Ridge National Laboratory, Oak Ridge, TN, United States; ^5^ Department of Molecular and Structural Biochemistry, North Carolina State University, Raleigh, NC, United States

**Keywords:** disorder, expression, kinetics, mutagenesis, transglycosylation, Hamamotoa singularis

## Abstract

*Hamamotoa (Sporobolomyces) singularis* codes for an industrially important membrane bound ß-hexosyltransferase (BHT), (BglA, UniprotKB: Q564N5) that has applications in the production of natural fibers such as galacto-oligosaccharides (GOS) and natural sugars found in human milk. When heterologously expressed by *Komagataella phaffii* GS115, BHT is found both membrane bound and soluble secreted into the culture medium. *In silico* structural predictions and crystal structures support a glycosylated homodimeric enzyme and the presence of an intrinsically disordered region (IDR) with membrane binding potential within its novel N-terminal region (1–110 amino acids). Additional *in silico* analysis showed that the IDR may not be essential for stable homodimerization. Thus, we performed progressive deletion analyses targeting segments within the suspected disordered region, to determine the N-terminal disorder region’s impact on the ratio of membrane-bound to secreted soluble enzyme and its contribution to enzyme activity. The ratio of the soluble secreted to membrane-bound enzyme shifted from 40% to 53% after the disordered N-terminal region was completely removed, while the specific activity was unaffected. Furthermore, functional analysis of each glycosylation site found within the C-terminal domain revealed reduced total secreted protein activity by 58%–97% in both the presence and absence of the IDR, indicating that glycosylation at all four locations is required by the host for the secretion of active enzyme and independent of the removed disordered N-terminal region. Overall, the data provides evidence that the disordered region only partially influences the secretion and membrane localization of BHT.

## Introduction

*Hamamotoa (Sporobolomyces) singularis* (*H. singularis*) expresses, under inducible conditions, a unique extracellular membrane-bound glycosylated ß-hexosyltransferase (BHT) (Gorin et al., 1964a; Gorin et al., 1964b). This distinctive membrane-bound enzyme has maintained biological interest for the last 60 years and is known by many names (BglA, UniprotKB: Q564N5) (Phaff and Carmo-Sousa, 1962; Gorin et al., 1964a; Gorin et al., 1964b; Gorin et al., 1964b; Blakely and MacKenzie, 1969; Ishikawa et al., 2005). In nature, *H. singularis* BHT functions as a glucosyl hydrolase that catalyzes the hydrolysis of cellobiose ß-(1–4) glycosidic linkages. Interestingly, BHT also has ß-galactosidase activity, demonstrated by its ability to cleave lactose (*β*-D-galactopyranosyl-(1→4)-*α*-D-glucopyranose). However, unlike ß-galactosidases, *H. singularis* BHT simultaneously carries out hydrolase and galactosyl transferase activities, converting lactose (independent of initial lactose concentration) to galacto-oligosaccharides (GOS) without extracellular accumulation of galactose (Gorin et al., 1964a; Gorin et al., 1964b; Blakely and MacKenzie, 1969) and to natural sugars found in human milk (Arnold et al., 2021b). These fibers are regarded as prebiotic components that have physiological effects on the make-up and functioning of the gut microbiota, thereby benefiting the health of the host (Torres et al., 2010; Azcárate-Peril et al., 2011; Bruno-Barcena and Azcarate-Peril, 2015; Monteagudo-Mera et al., 2016; Arnold et al., 2018; Panesar et al., 2018; Watson et al., 2019; Whittington et al., 2019; Arnold et al., 2021a; Arnold et al., 2021b). Due to its numerous health benefits, GOS are widely utilized as functional food additives on a global scale (Spherix Consulting Inc, 2010).

The 594-residue polypeptide that makes up the enzyme (BHT) has two distinct regions: the C-terminal domain, which is homologous to other glycosyl hydrolase family 1 (GH1) members, and the N-terminal section (residues 1–110), which is unique to BHT and has no homology to any known glycosyl transferases or β-glucosidases (Dagher et al., 2013; Dagher and Bruno-Bárcena, 2016). We previously showed the presence of an active 1–22 signal sequence with a membrane anchor signature inside the 110 N-terminal region using *in silico* analysis and subsequent functional studies using *K. phaffii* GS115 for secretion (Dagher and Bruno-Bárcena, 2016). In that study, the 1–22 signal sequence was replaced with MFα, which resulted in a 10-fold increase in the amount of secreted catalytically active rBHT in the culture broth compared to expression of full-length rBHT which remained membrane-bound. Surprisingly, the bulk of rBHT remained affixed to the *K. phaffii* GS115 membrane rather than being fully transferred to the medium (Dagher et al., 2013; Dagher and Bruno-Bárcena, 2016).

*In silico* analysis also revealed that the N-terminal region comprises regions of low complexity that have yet to be defined and characterized (Dagher et al., 2013; Dagher and Bruno-Bárcena, 2016). Furthermore, the crystal structures solved by Uehara et al., 2020 (HsBglA, PDB: 6M4E) (Uehara et al., 2020) and in this study (BHT, PDB: 7L74) showed a potential for an intrinsically disordered region (IDR) within the N-terminus. IDRs are flexible and extended protein segments known to lack organized secondary structure under physiological conditions. However, their biological function depends on this unstructured state (Uversky, 2019). Intrinsically disordered proteins (IDPs) exist in interchanging conformations rather than adapting well-defined structures as previously reviewed (Uversky, 2019). This is consistent with IDRs’ functional advantages and ability to fold in response to partner contact or in a template-dependent manner (Darling and Uversky, 2018).

It has been demonstrated that proteins with large stretches of IDRs are essential elements for membrane interactions because these flexible areas allow for protein-protein or protein-lipid interactions, great selectivity and low affinities for key components of signal transduction cascades (Cornish et al., 2020). Additionally, membrane attachment constricts the protein’s search space, consequently membrane localization can increase the effective concentration while simultaneously acting as a steric barrier to prevent interactions from occurring in solution (Cornish et al., 2020).

Proteome-wide investigations have shown connections between IDRs and several post-translational modifications (PTMs), including acetylation, methylation, and glycosylation (Gao and Xu, 2012). Previous studies by us and others in which *E. coli* was unable to express active rBHT suggested the critical importance of PTMs for appropriate folding and/or enzymatic activity (Ishikawa et al., 2005; Dagher and Bruno-Bárcena, 2016). One of the most important post-translational modifications of proteins is glycosylation, which primarily involves the attachment of glycans to the nitrogen atom of asparagine residues (N-linked) or to the hydroxyl oxygen of serine, threonine, or tyrosine residues (O-linked). Other important post-translational modifications of proteins include C-mannosylation, phospho-serine glycosylation, and glypiation (formation of GPI anchors) (Prabakaran et al., 2012; Darling and Uversky, 2018). In *K. phaffii* GS115, N-glycans form high-mannose-type heterogeneous oligosaccharides beginning with the addition of the core unit Glc_3_Man_9_GlcNAc_2_ (Glc = glucose; GlcNAc = N-acetylglucosamine; Man = mannose) at asparagine in the recognition sequence Asn-X-Ser/Thr X≠P (Bretthauer and Castellino, 1999). N-glycosylation has been shown to influence enzymatic activity, stability, and cell surface expression as previously reviewed (Ge et al., 2018).

Further investigations into the intricacies of the structure of this enzyme are therefore needed to provide suggestions on how to enhance soluble secretion of rBHT. In this study we conducted a detailed kinetic analysis of rBHT variants lacking progressive portions of the IDR, in comparison to the full-length enzyme. To evaluate the impacts on protein secretion and enzyme activity, this study looked at modifications in the IDR length, N-glycosylation sites, and dimer stability. The results provide insight into the dynamics of the IDR related to enzyme secretion and localization of active rBHT generated by *K. phaffii* GS115.

## Materials and methods

### Strains and media

The bacterial and *K. phaffii* GS115 strains used in this study are shown in Table 1. Bacteria were grown at 37°C in Luria-Bertani (LB) medium with antibiotic ampicillin (100 μg/mL) (Thermo Fisher Scientific). Growth and maintenance of GS115 (Invitrogen Life Technologies, Thermo Fisher Scientific) was described previously (Dagher and Bruno-Bárcena, 2016). *E. coli* XL1-Blue was used as the cloning host (Agilent Technologies, Thermo Fisher Scientific). The plasmid pPIC9 (Invitrogen Life Technologies, Thermo Fisher Scientific) was used as cloning vector containing codon optimized *Bht* (*rBht* sequences) (GenBank accession number JF29828).

**TABLE 1:** Strains and plasmids used in this study.

*Strains/Plasmids*	^a^ *Description or genotype*	*Source or Reference*
*E. coli*		
XL1-Blue	*recA1 endA1 gyrA*96 *thi-1 hsdR17 supE44 relA1 lac* [F′ *pro*AB *lacI* ^q^ZΔM15 Tn*10* (Tet^R^)]	Agilent
*K. phaffii* GS115		
GS115	*his4* (his^−^ mut^+^)	Invitrogen
JB210	GS115::*MFα*-*rBht*_(1–594)_-*HIS* (his^+^ mut^+^)	Dagher and Bruno-Bárcena (2016)
JB212	GS115::*MFα*-*rBht*_(23–594)_-*HIS* (his^+^ mut^+^)	Dagher and Bruno-Bárcena (2016)
JB216	GS115::*MFα-rBht*_(111–594)_-*HIS* (his^+^ mut^+^)	Dagher and Bruno-Bárcena (2016)
JB223	GS115::MFα-*rBht*_(32–594)_-*HIS* (his^+^ mut^+^)	This study
JB224	GS115::*MFα-rBht*_(54–594)_-*HIS* (his^+^ mut^+^)	This study
JB225	GS115::*MFα*-*rBht*_(57–594)_-*HIS* (his^+^ mut^+^)	This study
JB226	GS115::*MFα-rBht*_(82–594)_-*HIS* (his^+^ mut^+^)	This study
JB227	GS115::*MFα-rBht*_(95–594)_-*HIS* (his^+^ mut^+^)	This study
JB228	GS115::*MFα-rBht*_(103–594)_-*HIS* (his^+^ mut^+^)	This study
JB229	GS115::*MFα*-*rBht*_(23–594) (N289Q)_-*HIS* (his^+^ mut^+^)	This study
JB230	GS115::*MFα*-*rBht*_(23–594) (N297Q)_-*HIS* (his^+^ mut^+^)	This study
JB231	GS115::*MFα*-*rBht*_(23–594) (N431Q)_-*HIS* (his^+^ mut^+^)	This study
JB232	GS115::*MFα-rBht_(23–594) (N569Q)_-HIS* (his^+^ mut^+^)	This study
JB238	GS115::*MFα-rBHT_(57-594)(N289Q)_-HIS* (his^+^ mut^+^)	This study
JB239	GS115::*MFα-rBHT_(57-594)(N297Q)_-HIS* (his^+^ mut^+^)	This study
JB240	GS115::*MFα-rBHT_(57-594)(N431Q)_-HIS* (his^+^ mut^+^)	This study
JB241	GS115::*MFα-rBHT_(57-594)(N569Q)_-HIS* (his^+^ mut^+^)	This study
JB242	GS115::pPIC9 (his^+^ mut^+^) control	This study
*Plasmids*		
pPIC9	*K. phaffii* GS115 integrative vector carrying *AOX1* promoter and transcription terminator, *HIS4*, *Amp* ^*r*^ in *E. coli,* pBR322 ori, alpha factor pre-pro leader from *S. cerevisiae* (*MFα*)	Invitrogen
pJB110	pPIC9-*MFα-rBht*_(1–594)_-*HIS*	Dagher and Bruno-Bárcena (2016)
pJB112	pPIC9-*MFα-rBht*_(23–594)_-*HIS*	Dagher and Bruno-Bárcena (2016)
pJB116	pPIC9-*MFα*-*rBht*_(111–594)_-*HIS*	Dagher and Bruno-Bárcena (2016)
pJB123	pPIC9-*MFα*-*rBht*_(32–594)_-*HIS*	This study
pJB124	pPIC9-*MFα*-*rBht*_(54–594)_-*HIS*	This study
pJB125	pPIC9-*MFα*-*rBht*_(57–594)_-*HIS*	This study
pJB126	pPIC9-*MFα*-*rBht*_(82–594)_-*HIS*	This study
pJB127	pPIC9-*MFα*-*rBht*_(95–594)_-*HIS*	This study
pJB128	pPIC9-*MFα*-*rBht*_(103–594)_-*HIS*	This study
pJB129	pPIC9-*MFα-rBht*_(23–594) (N289Q)_-*HIS*	This study
pJB130	pPIC9-*MFα-rBht*_(23–594) (N297Q)_-*HIS*	This study
pJB131	pPIC9-*MFα-rBht*_(23–594) (N431Q)_-*HIS*	This study
pJB132	pPIC9-*MFα-rBht*_(23–594) (N569Q)_-*HIS*	This study
pJB138	pPIC9-*MFα-rBht*_(57–594) (N289Q)_-*HIS*	This study
pJB139	pPIC9-*MFα-rBht*_(57–594) (N297Q)_-*HIS*	This study
pJB140	pPIC9-*MFα-rBht*_(57–594) (N431Q)_-*HIS*	This study
pJB141	pPIC9-*MFα-rBht*_(57–594) (N569Q)_-*HIS*	This study

^a^ *MFα*, *Saccharomyces cerevisiae* alpha factor pre-pro secretion leader found in pPIC9 vector is indicated in constructions.

### Plasmid constructions, expression, and purification of rBHT-truncated variants

All molecular biology protocols were carried out as previously described (Dagher and Bruno-Bárcena, 2016). Briefly, expression by *K. phaffii* GS115 was achieved by homologous integration of DNA fragments bearing *rBht* sequences, for example, coding for mutations and truncations.

The truncated *rBht* sequences were generated by PCR amplification using pJB110 (pPIC9*-MFα*-*rBht*_*(1–594)*_-*HIS*) as template. Primers were purchased from Integrated DNA Technologies (IDT Coralville, IA, USA) (listed in Table 2). When appropriate, the primers included restriction sites to facilitate cloning (Table 2). Briefly, primer pairs for sequences coding for the N-terminal truncated *rBht* sequences encoding protein sizes included the following: 23–594 (primers: JBB7/JBB5), 32–594 (primers: JBB21/JBB5), 54–594 (primers: JBB22/JBB5), 57–594 (primers: JBB23/JBB5), 82–594 (primers: JBB24/JBB5), 95–594 (primers: JBB25/JBB5) and 103–594 (primers: JBB26/JBB5). PCR amplicons were digested with *Xho*I-*Not*I and cloned into pPIC9 (Invitrogen Life Technologies, Thermo Fisher Scientific) generating pJB112 (pPIC9*-MFα*-*rBht*_*(23–594)*_-*HIS*), pJB123 (pPIC9*-MFα*-*rBht*_*(32–594)*_-*HIS*), pJB124 (pPIC9*-MFα*-*rBht*_*(54–594)*_-*HIS*), pJB125 (pPIC9*-MFα*-*rBht*_*(57–594)*_-*HIS*), pJB126 (pPIC9*-MFα*-*rBht*_*(82–594)*_-*HIS*), pJB127 (pPIC9*-MFα*-*rBht*_*(95–594)*_-*HIS*) and pJB128 (pPIC9*-MFα*-*rBht*_*(103–594)*_-*HIS*) respectively.

**TABLE 2:** Primers, antibodies, and substrates used in this study.

*Name*	*Primer*	*^a^Sequence*	*Source*
JBB5	*Not*I*-rBht*-6X*HIS* Reverse	5′-aag​gaa​aaa​aGC​GGC​CGC​TTA​GTG​GTG​GTG​GTGG​TGG​TGC​AGA​TGA​TTA​CGC​CCA​AAT​TG - 3′	Dagher and Bruno-Bárcena (2016)
JBB7	*Xho*I-*MFα*-*rBht*_*(23–594)*_ Forward	5′-ccg​CTC​GAG​AAA​AGA​GAG​GCT​GAA​GCT​GTT ACTTATCCGGGAGCC- 3′	Dagher and Bruno-Bárcena (2016)
JBB21	*Xho*I-*MFα*-*rBht*_*(32–594)*_ Forward	5′- GAA​GAA​GGG​GTA​TCT​CTC​GAG​AAA​AGA​GAGG​CTG​AAG​CTT​CCC​TGA​CGA​GCA​ATT​ACG - 3′	This study
JBB22	*Xho*I-*MFα*-*rBht*_*(54–594)*_ Forward	5′- GAA​GAA​GGG​GTA​TCT​CTC​GAG​AAA​AGA​GAGGC​TGA​AGC​TAC​CGG​TAC​AGC​AGA​ATT​AG - 3′	This study
JBB23	*Xho*I-*MFα*-*rBht*_*(57–594)*_ Forward	5′-GAA​GAA​GGG​GTA​TCT​CTC​GAG​AAA​AGA​GAGGC​TGA​AGC​TGC​AGA​ATT​AGA​TGC​GCT​GTG - 3′	This study
JBB24	*Xho*I-*MFα*-*rBht*_*(82–594)*_ Forward	5′- GAA​GAA​GGG​GTA​TCT​CTC​GAG​AAA​AGA​GAGGC​TGA​AGC​TAC​AGT​GCC​CGA​TGA​TTA​TAA​G - 3′	This study
JBB25	*Xho*I-*MFα*-*rBht*_*(95–594)*_ Forward	5′- GAA​GAA​GGG​GTA​TCT​CTC​GAG​AAA​AGA​GAG​GCTG​AAG​CTA​GTT​ATG​CAT​TAG​CAG​GGT​ATG - 3′	This study
JBB26	*Xho*I-*MFα*-*rBht*_*(103–594)*_ Forward	5′- GAA​GAA​GGG​GTA​TCT​CTC​GAG​AAA​AGA​GAG​GCTG​AAG​CTA​CAA​GCG​AGA​TTG​CCG​GAC - 3′	This study
JBB27	*BHT*_*(N289Q)*_ Forward	5′- TCA​CAA​AAT​AGT​GGT​CTG​CCA​TAC​CAG ***C*** T ***T*** AC ***G*** TAT​CCA​GAA​GGT​ATT​AAC​AG - 3′	This study
JBB28	*BHT*_*(N289Q)*_ Reverse	5'- CTG​TTA​ATA​CCT​TCT​GGA​TA ***C*** GT ***A*** A ***G*** CTG​GTATG​GCA​GAC​CAC​TAT​TTT​GTG​A - 3′	This study
JBB29	*BHT*_*(N297Q)*_ Forward	5'- CAA​TCT​GAC​GTA​TCC​AGA​AGG ***G*** AT ***CC*** A ***G*** AG CACCTCCGCTG - 3′	This study
JBB30	*BHT*_*(N297Q)*_ Reverse	5'- CAG​CGG​AGG​TGC​T ***C*** T ***GG*** AT ***C*** CCT​TCT​GGA​TAC GTCAGATTG - 3′	This study
JBB31	*BHT*_*(N431Q)*_ Forward	5'- CGG​GAT​CGC​GAA​TTG​TAT​TCG​C ***C*** A ***G*** CAA ***TCG*** GA ***T*** CCG​AAT​TGG​CCA​GTG​TGT​GAA​G - 3′	This study
JBB32	*BHT*_*(N431Q)*_ Reverse	5'- CTT​CAC​ACA​CTG​GCC​AAT​TCG​G ***A*** TC ***CGA*** TTG ***C*** T ***G*** GCG​AAT​ACA​ATT​CGC​GAT​CCC​G - 3′	This study
JBB33	*BHT*_*(N569Q)*_ Forward	5'- GAA​ATT​CGG​ATT​TCA​GTT​TGT​T ***C*** A ***G*** CAA​TC ***G*** G ATCCCGATCTGACAC - 3′	This study
JBB34	*BHT*_*(N569Q)*_ Reverse	5'- GTG​TCA​GAT​CGG​GAT​C ***C*** GAT​TG ***C*** T ***G*** AAC​AA ACTGAAATCCGAATTTC - 3′	This study
JBB3	*rBht* Forward internal sequencing	5´ - ATC​ACT​ATG​CCA​GCA​CGC​AGT​GTA - 3′	Dagher et al. (2013)
JBB4	*rBht* Reverse internal sequencing	5´ - TTT​AAA​GCC​GAT​TTC​ACC​TGC​CGC - 3′	Dagher et al. (2013)
5′ AOX1	AOX1	5′- GAC​TGG​TTC​CAA​TTG​ACA​AGC - 3′	Invitrogen
3′ AOX1	AOX1	5′- GCA​AAT​GGC​ATT​CTG​ACA​TCC - 3′	Invitrogen
α-factor	*MFα*	5´ - TAC​TAT​TGC​CAG​CAT​TGC​TGC - 3′	Invitrogen
*Antibodies*	*Antigen*		
Mouse anti-HIS	6XHIS		GenScript
*Substrates*	*Abbreviation*		
*o*NP-β-D-gluco-pyranoside	ONP-Glc		Sigma

^a^ Coding regions are capitalized, mutated nucleotides are bold and italicized, *MFα, Saccharomyces cerevisiae* alpha factor pre-pro secretion leader sequence.

Site directed mutagenesis was performed using complementary oligonucleotides designed to incorporate the desired base changes using QuickChange site directed mutagenesis kit (Agilent Technologies Santa Clara, CA, USA) according to manufacturer’s instructions. The generated variants include single amino acid exchanges replacing asparagine for glutamine in putative N-glycosylation sites using as template pJB112 (pPIC9-*MFα*-*rBht*_*(23–594)*_-*HIS*). The residues modified include positions; N289Q (primers: JBB27/JBB28) to generate pJB129 (pPIC9-*MFα-rBht*_(23–594) (N289Q)_-*HIS*), N297Q (primers: JBB29/JBB30) to generate pJB130 (pPIC9-*MFα-rBht*_(23–594) (N297Q)_-*HIS*), N431Q (primers: JBB31/JBB32) to generate pJB131 (pPIC9-*MFα-rBht*_(23–594) (N431Q)_-*HIS*), and N569Q (primers: JBB33/JBB34)) to generate pJB132 (pPIC9-*MFα-rBht*_(23–594) (N569Q)_-*HIS*) (Dagher and Bruno-Bárcena, 2016) (Table 1). Next, the set of primers JBB23/JBB5 were used on each sequence to obtain plasmids pJB138 (pPIC9-*MFα-rBht*_(57–594) (N289Q)_-*HIS*), pJB139 (pPIC9-*MFα-rBht*_(57–594) (N297Q)_-*HIS*), pJB140 (pPIC9-*MFα-rBht*_(57–594) (N431Q)_-*HIS*) and pJB141 (pPIC9-*MFα-rBht*_(57–594) (N569Q)_-*HIS*).

As described above PCR amplicons were digested with *Xho*I-*Not*I and cloned into pPIC9 (Invitrogen Life Technologies, Thermo Fisher Scientific). DNA fragments from restriction enzyme digests were purified from agarose gels using QIAquick gel extraction kit (Qiagen, Hilden, Germany). All mutations were confirmed with restriction digests for detecting restriction sites in primers and by Sanger sequencing performed by the Azenta Life Sciences (USA) using primers JBB3, JBB4, 5′ AOX1, 3’ AOX1 and α-factor (Table 2).

### *K. phaffii* GS115 transformation and expression

*K. phaffii* GS115 was transformed with linearized plasmids as per the Invitrogen *Pichia* Expression Kit manual (Invitrogen, USA). Plasmid integration and Mut^+^ phenotype in histidine positive colonies was confirmed by sequencing PCR products generated by primers 5′ AOX1 and 3’ AOX1(Invitrogen Pichia expression kit). Single copy integration was confirmed as previously described (Dagher and Bruno-Bárcena, 2016).

Expression and purification have been described previously (Dagher and Bruno-Bárcena, 2016). Briefly, filtered culture media was purified using the ÄKTApurifier and HISTrap™ HP Nickel column (GE Healthcare, Life sciences). The purified proteins were quantified by Bradford protein assay (Thermo Fisher Scientific) (Bradford, 1976).

### SDS-PAGE and Western immunoblot analysis

Proteins were analyzed by SDS-PAGE using 10% resolving gels and visualized using Coomassie and silver stains (Bio-Rad, Hercules, CA). Immunoblots were probed with 1:10,000 dilution of anti-HIS antibody (GenScript, Piscataway, NJ) followed by 1:10,000 dilution of alkaline phosphatase conjugated goat anti-mouse antibody (GenScript, Piscataway, NJ). Detection was carried out with 1-Step™ NBT/BCIP Substrate Solution according to manufacturer’s instructions (Thermo Fisher Scientific).

### Enzyme assays

Hydrolysis of o-nitrophenyl-β-D-glucopyranoside (ONP-Glc) was followed by measurement of absorbance at 405 nm for determination of β-glucosidase activity using the methods described previously (Dagher and Bruno-Bárcena, 2016). Briefly, cells were harvested by centrifugation (5,000 g at 4°C), to separate soluble rBHT from membrane bound rBHT. The cells were then washed two times with 50 mM phosphate-citrate buffer (pH 5). Assays on soluble secreted and membrane bound rBHT were performed in a 50 mM phosphate-citrate buffer under optimal temperature of 42°C and optimal pH 5 for 10 min. Reactions were stopped by the addition of an equal volume of 0.25 M sodium carbonate and the absorbance was measured at 405 nm.

The Michaelis-Menten constants (Km and Vmax) of 0.3 µg rBHT (at 42°C) were determined by varying ONP-Glc from 0 to 10.4 mM in 50 mM phosphate-citrate buffer (pH 5) and measuring the initial reaction rate at 20°C, 30°C, 42°C, and 55°C. The kinetic constants at each temperature were determined with OriginPro 7.5 using nonlinear regression of the Hill equation with a Hill coefficient of 1.

### Secondary structure prediction

Secondary structure consensus prediction of BHT was performed at the PSIPRED server (protein structure prediction) (Jones, 1999; Cozzetto et al., 2016; Buchan and Jones, 2019) and at the NPS@server (network protein sequence analysis) (Combet et al., 2000). The signal sequence was predicted using the SignalP 5.0 algorithm (Almagro Armenteros et al., 2019). Protein disorder was predicted using the consensus of six methods, Dispred3 (Jones and Cozzetto, 2015), Phyre2 (Kelley et al., 2015), IUPred2A (Meszaros et al., 2018), PONDR-VSL2 (Peng et al., 2005) and GlobPlot (prediction of protein disorder and globularity) (Jones, 1999; Linding et al., 2003), PHYRE2 (Kelley et al., 2015). Domain boundaries were predicted using the DomPred server (Bryson et al., 2007) and Pfam version 32.0 (El-Gebali et al., 2018).

### N-glycosylation prediction

BHT N- and O-glycosylation site prediction was performed at the GlycoEP server (Chauhan et al., 2013).

### Phosphorylation site prediction

BHT phosphorylation site prediction was performed using DEPP (Disorder enhanced phosphorylation predictor), also known as DisPhos1.3 (http://www.dabi.temple.edu/disphos/) (Iakoucheva et al., 2004) and NetPhosYeast1.0 (http://www.cbs.dtu.dk/services/NetPhosYeast/) (Ingrell et al., 2007).

### Structural modeling programs

Structural figures and structural superimpositions were generated in PyMOL (http://www.schrodinger.com/pymol/) (Schrodinger, 2022). A homodimer is present in the crystal asymmetric unit; however, the monomer was considered for structural analysis.

### Crystallization

rBHT_(23–594)_-HIS was further purified by gel filtration chromatography on a Sephacryl S-300 (GE Healthcare, Life Sciences) column equilibrated with 100 mM Tris pH 7.5, 200 mM sodium chloride, 1 mM dithiothreitol to reduce aggregates and concentrated to 6 mg/mL using Amicon^®^ Ultra 15, molecular weight cut-off 10,000 (Millipore Sigma) in 10 mM HEPES pH 7.5. Protein concentrations were determined by Lowry method (Lowry et al., 1951) using bovine serum albumin as a standard. The crystals were grown by vapor diffusion using the sitting drop method. The crystals were grown using a crystallization solution made by mixing 1 µL (10 μg/μL) purified protein with 1 µL of precipitant solution (35% polyethylene glycol 4k, 0.1 M HEPES pH 7.5, 0.2 M calcium chloride) and equilibrating the drop against 0.5 mL of the precipitant at 22°C–23°C. Crystals usually appeared in less than a week. Prior to data collection, the crystals were soaked for 10 min in a cryoprotectant solution (35% polyethylene glycol 4k, 0.1 M HEPES pH 7.5, 0.2 M calcium chloride, 20% ethylene glycol) and then immediately flash-vitrified in liquid nitrogen.

### Data collection, processing, and structure refinement

Single crystal diffraction data were collected at the Life Sciences Collaborative Access Team facility (Advanced Photon Source sector 21, Argonne National Laboratory (Lemont, IL, USA) on beamline 21G (Table 3). The data covered 360° in 0.5-degree increments. The frames were integrated with XDS (Kabsch, 2010) and scaled with Aimless (Evans and Murshudov, 2013) in AutoProc (Vonrhein et al., 2011). The structure was solved by molecular replacement in PHENIX (Adams et al., 2010) using a homology model generated by RaptorX (Källberg et al., 2012). The structure was rebuilt and refined with PHENIX and then optimized with PDB-REDO (Joosten et al., 2014). Coot (Emsley et al., 2010) was used to add and to optimize individual residues, posttranslational modifications and ligands.

**TABLE 3:** Data collection and refinement statistics of the structure of rBHT_(23–594)_-HIS at pH 7.5.

Data set	
PDB CODE	7L74
Crystal	0.2 × 0.05 mm
Protein	rBHT_(23–594)_-HIS, S-300 pure in 10 mM HEPES pH7.5
Well	35% PEG4K, 0.1 M HEPES pH 7.5, 0.2 M CaCl_2_
Drop	1:1 Protein:Well
Cryoprotectant	35% PEG4K, 0.1 M HEPES pH 7.5, 0.2 M CaCl_2_, 20% ethylene glycol
Final pH	7.5
Data collection	
Space group	C2
Unit-cell parameters	a = 196.319
	b = 63.209
	c = 105.006
	α = 90.0
	β = 100.423
	γ = 90.0
Molecules/asymmetry unit (Chains per A.U.)	2
Matthews coefficient (Å^3^.Da^-1^)	2.46
Solvent content (%)	50.1
Resolution-high (Å)	2.25
Resolution-low (Å)	29.96
Beamline	APS 21-G
Wavelength (Å)	0.97856
Total number of observed reflections	56424
Completeness (%)	98.10 (90.38)
Average I/σ(I)	9.8 (2.5)
R_merge_^a^(%)	0.139 (0.604)
Refinement	
R_factor_	0.189 (0.265)
R_free_	0.230 (0.297)
Ave B-factor (Å^2^)	18.93
Number of atoms (Non-Hydrogen Atoms per A.U.)	9,355
Amino acid residues	541
Water molecules	523
Resolution used in refinement (Å)	2.25
R_work_^b^/R_free_^c^ (%)	
RMSD bond distance (Å)	0.009
RMSD bond angle (°)	1.310
RMSD Chiral	0.076

^a^R_merge_ = ∑_hkl_∑_I_ | I_i_(hkl) −<I(hkl)> | ∑_hkl_∑_i_I_i_(hkl), where I_i_(hkl) is the intensity of the ith measurement of reflection hkl, including symmetry-related reflections, and <I(hkl)> is their average. ^b^R_work_ = ∑_h_∑_i_ ||Fo| − |F_c_||/∑ |F_o_|. ^c^R_free_ is Rwork for ∼ 5% of the reflections that were excluded from the refinement.

## Results

### Crystal structure of rBHT shows hallmarks of intrinsic disorder in the N-terminal domain

When expressed by *K. phaffii* GS115, the rBHT variant (rBHT_(23–594)_-6XHIS) is functionally independent of its location either associated with the membrane or soluble (Dagher et al., 2013; Dagher and Bruno-Bárcena, 2016). To gain insights into the structure-function characteristics, we used soluble rBHT_(23–594)_-6XHIS to solve the crystal structure. Data processing and refinement statistics are presented in Table 3 and the final model was deposited in the Protein Data Bank (PDB: 7L74). The structure was solved by molecular replacement at a resolution of 2.25 Å and the asymmetric unit contains two molecules of rBHT_(23–594)_-6XHIS and the value *V*_m_ was estimated to be 2.46 Å^3^.Da^-1^.

rBHT folds into two domains, the N-domain, and the C-domain. The structure for enzymatic activity in the C-terminal region is composed of a sugar-binding catalytic domain organized in a (α/β)_8_ TIM barrel that stretches from residue 116 to residue 547 (BHT, PDB: 7L74). Eight parallel β-strands comprise the core BHT (α/β)_8_ barrel, which is coupled to eight external α-helices that is common to Glycoside Hydrolase Family I (GH1) members (Henrissat et al., 1995; Glycoside Hydrolase Family, 2012).

The initial portion of the N-domain (residues 23–53) upstream of the carboxy GH1 domain, lacked electron density and could not be modelled indicating the presence of a potential N-terminal intrinsically disordered region (IDR). Additional support for the IDR within the N-terminal domain comes from a previously solved crystal structure (HsBglA, PDB: 6M4E) (Uehara et al., 2020). HsBglA and rBHT crystal structures show minor structural differences with an RMSD across all Cα pairs of 0.22 Å.

### BHT N-terminal IDR composition

The relevance of the disordered regions in membrane lipid association and interactions of membrane associated proteins can only be understood by examining the properties of the interacting environment (Mohammad et al., 2019; Cornish et al., 2020; Csizmadia et al., 2021). This can be complicated by the multiple interactions and functions exhibited by disordered regions (Theillet et al., 2013; Uversky, 2019) and their ability to fold upon contact in a template-dependent manner or with specific ligand partners (Bürgi et al., 2016).

**FIGURE 1:**
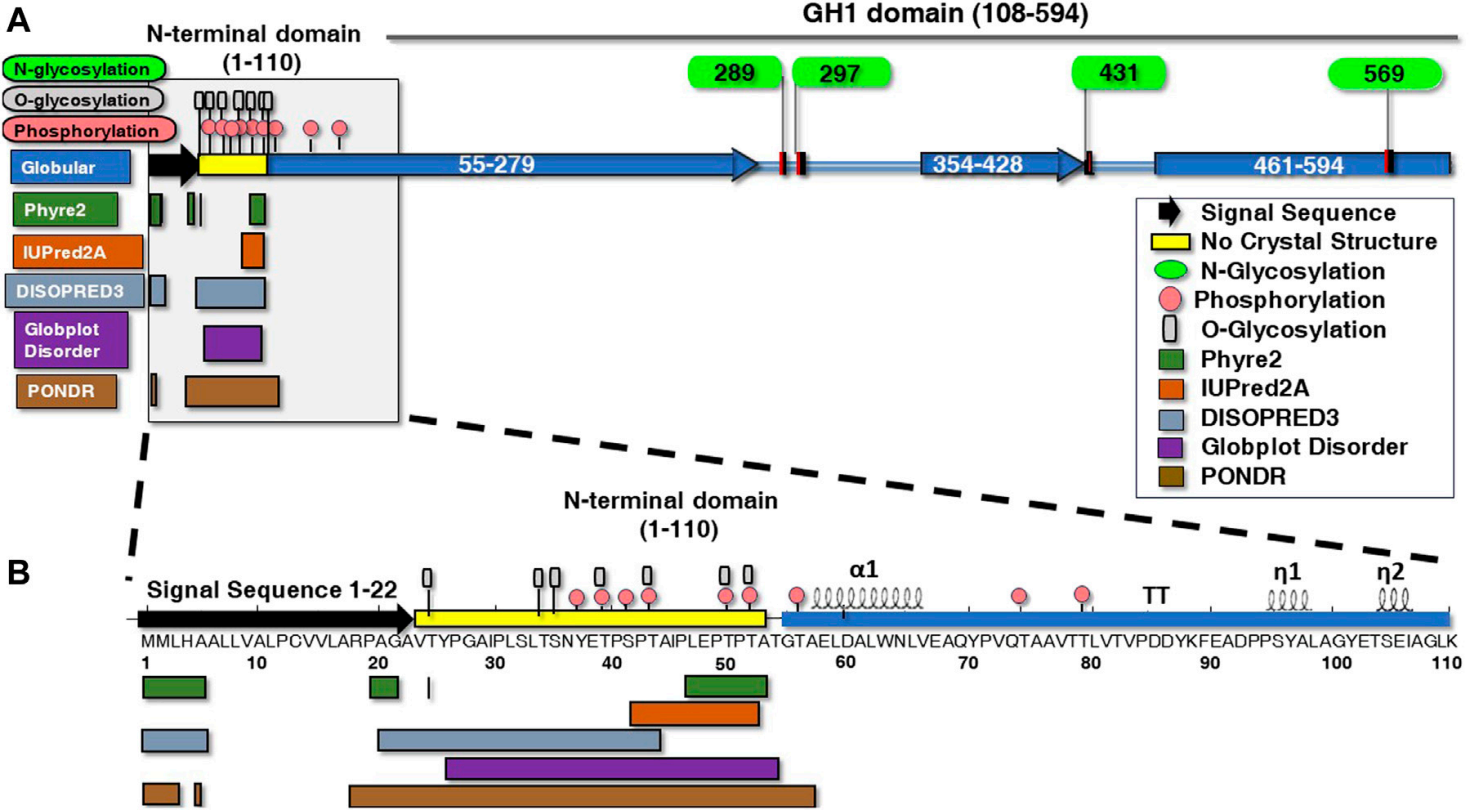
Structural posttranslational modifications and disordered versus ordered secondary motifs of ß*-*hexosyltransferase from *H. singularis.* **(A)** BHT protein glycosylation, phosphorylation and secondary structures were predicted in the amino terminus using various algorithms. Depicted are the structural elements, conserved regions, and functional domains of BHT using PSIPRED and Globplot Globular prediction tools (blue rectangles). GlycoEP displays N-glycosylation (green ovals) (N289, N297, N431, N569). **(B)** The N-terminal domain (1–110) has been expanded along with predicted glycosylation, phosphorylation, and secondary structures. Numbers indicate BHT amino acid residue number. The secondary structure elements of BHT shown above the amino acid residues was generated with *ENDscript* (Robert and Gouet, 2014) (https://endscript.ibcp.fr). Disordered regions within the N-terminal domain were predicted using algorithms Phyre2 (1–5, 20–21, 24, 47–53), IUPRED2A (42–52), DISOPRED3 (1–5, 21–43) Globplot Disorder (26–54), and PONDR (1–3, 5, 18–57). Phosphorylation servers DisPhos1.3 and NetPhosYeast1.0 predicted phosphorylation sites within the disordered region (pink circles) (37, 39, 41, 43, 50, 52). GlycoEP displays O-glycosylation (gray rectangles) (24, 34, 35, 39, 43, 50, 52), while no N-glycosylation or C-mannosylation sites were predicted.

To make the IDR structure accessible for systematic analysis, IDR boundaries and PTM predictions needed to be made to reveal incomplete regions, which is particularly important for IDR analysis as described and shown below (Figure 1). This compelled us to perform a series of *in silico* structural predictions by using five available prediction tools over the full length of BHT (Figure 1). By combining different disorder predictors we expect to reinforce the reliability of the predicted regions since they use different definitions of disorder (Lieutaud et al., 2016). For example, PSIPRED and Globplot methods were employed to strengthen the lack of secondary structure and globular domains in the IDR region. Upon comparison, the disorder datasets derived from Phyre2, IUPred2A, DISOPRED3, Globplot Disorder and PONDR indicate probable disorder boundaries throughout the unique 1–110 N-terminal region and a common overlapping boundary at residues 52–53 (Figure 1), in agreement with PDB: 7L74 N-terminal boundary lacking electron density.

IDRs often contain a substantial degree of post-translational modifications (PTMs) such as phosphorylation, glycosylation, ubiquitination, acylation, and others that mediate potential interactions with high specificity (van der Lee et al., 2014; Cornish et al., 2020). For instance, phosphorylation can stabilize the tertiary structural organization of the IDR while enhancing and stabilizing its binding to the protein’s ligand (Gsponer et al., 2008; Nishi et al., 2011). Bioinformatic analysis has suggested that this function is tunable by PTMs and correlated with a high content of serine, threonine, glutamine and asparagine (Chuang et al., 2020).

The BHT IDR (resides 23–53) is composed of 29.1% serine and threonine residues that may act as potential phosphorylation/O-glycosylation sites. This finding is significant since phosphorylation is thought to function as an electrostatic switch by reducing the net charge thereby reducing membrane interactions (Aivazian and Stern, 2000; Cornish et al., 2020). O-linked glycosylation is also predicted to populate the BHT IDR (T24, T34, S35, T39, T43, T50, T52) (Figure 1B) where it likely functions along with phosphorylation (Y37, T39, S41, T43, T50, T52) to protect the region from proteolysis (Nishikawa et al., 2010; Prates et al., 2018).

It is known that disordered proteins often display a compositional bias toward polar residues and depleted of hydrophobic amino acids (Uversky, 2019). The disorder promoting residues are known to include aspartic acid, methionine, lysine, arginine, serine, glutamine, glycine, alanine, proline, and glutamic acid and commonly found on the surface of proteins (Theillet et al., 2013; Uversky, 2019). The BHT disordered region (residues 23–53) is made up of 3.2% valine, 9.7% alanine, 6.5% isoleucine, 9.7% leucine, 6.5% tyrosine, 3.2% glycine, 22.6% proline, 3.2% asparagine, 6.5% glutamic acid, 9.7% serine, and 19.4% threonine (Figure 1B). Surprisingly, hydrophobic amino acids make up 35.6% of the BHT IDR (Figure 1B).

### Expression and secretion of truncated N-terminal rBHT variants by *K. phaffii* GS115

Biologically important disordered regions have also been known as N-terminal fusion carriers to promote protein folding, act in folding quality control and thus enhance protein solubility. Our approach was to utilize the *in silico* analysis (Figure 1B) to perform progressive and selective deletions of the predicted IDR and to determine their impact on soluble secretion of catalytically active rBHT. Predictions were also made by the algorithm DisPhos1.3 (DEPP) that uses disorder information to help improve and discriminate between phosphorylation and non-phosphorylation sites (Materials and Methods). Furthermore, the accuracy of DEPP reaches 76.0±0.3%, 81.3±0.3% and 83.3±0.3% for serine, threonine, and tyrosine respectively (Iakoucheva et al., 2004; Ingrell et al., 2007).

**FIGURE 2:**
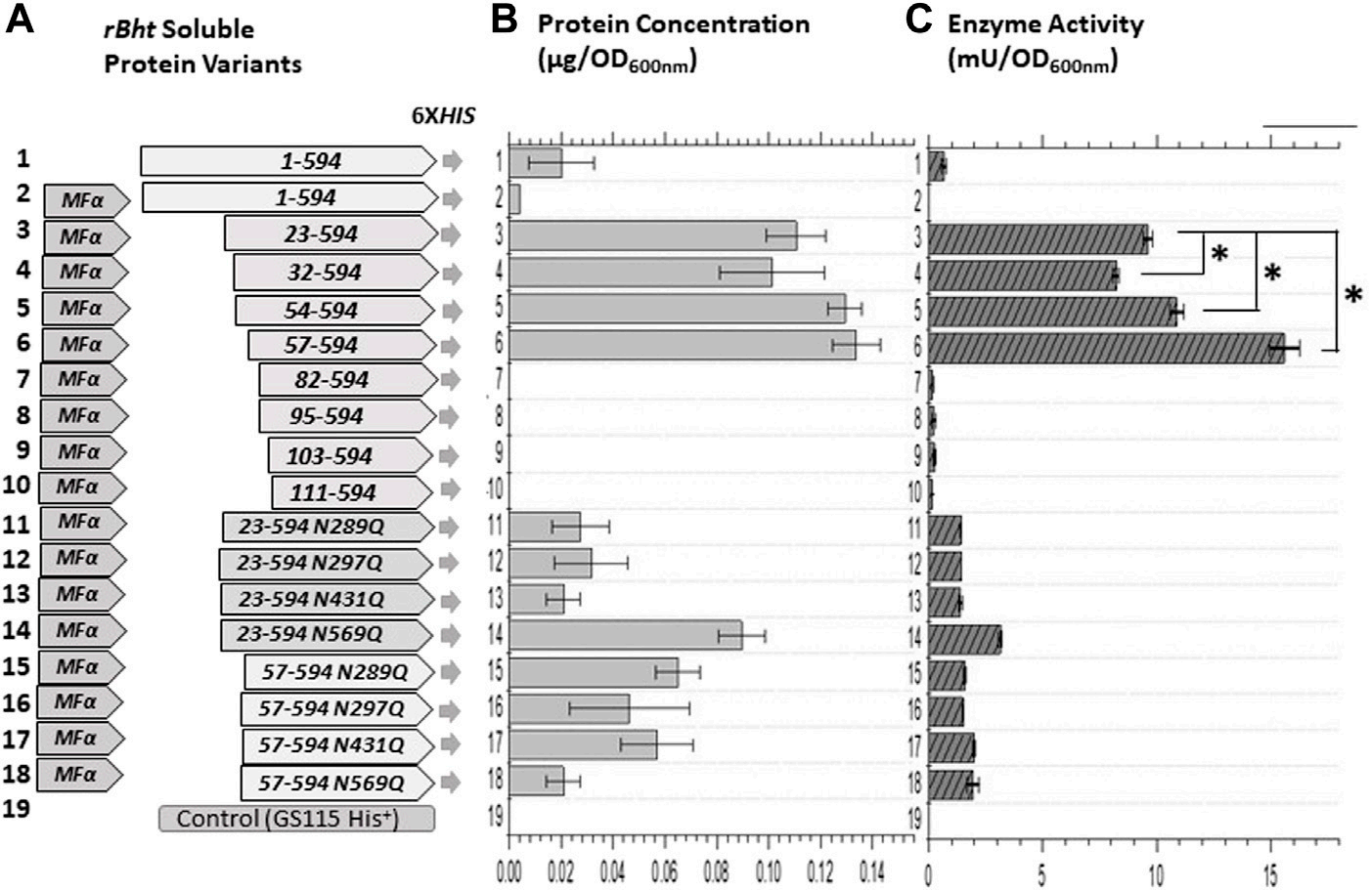
Enzyme activity comparisons of *K. phaffii* GS115 strains carrying sequences of *rBht*-*HIS* under the *AOX1* promoter. **(A)** Graphic representations of chimeric genes generated containing combinations of leader domains and ORFs of *rBht-HIS* sequences. Specific tags, mutations and deletions are indicated. **(B)** Protein concentration of soluble secreted protein normalized for the final culture (OD_600nm_). **(C)** Enzymatic activity of the secreted protein normalized for the final culture (OD_600nm_). The following recombinant strains were compared: row 1, GS115::*rBht*_(1–594)_-*HIS*; row 2, GS115::*MFα*-*rBht*_(1–594)_-*HIS*; row 3, GS115::*MFα*-*rBht*_(23–594)_-*HIS*; row 4, GS115::*MFα-rBht*_(32–594)_-*HIS*; row 5, GS115::*MFα*-*rBht*_(54–594)_-*HIS*; row 6,GS115::*MFα*-*rBht*_(57–594)_-*HIS*; row 7, GS115::*MFα*-*rBht*_(82–594)_-*HIS*; row 8, GS115::*MFα*-*rBht*_(95–594)_-*HIS*; row 9, GS115::*MFα*-*rBht*_(103–594)_-*HIS*; row 10, GS115::*MFα*-*rBht*_(111–594)_-*HIS*; row 11, GS115::*MFα*-*rBht*_(23–594)_ (N289Q)-*HIS*; row 12, GS115::*MFα*-*rBht*_(23–594)_ (N297Q)-*HIS*; row 13, GS115::*MFα*-*rBht*_(23–594)_ (N431Q)-*HIS;* row 14*,* GS115::*MFα*-*rBht*_(23–594)_ (N569Q)-*HIS;* row 15, GS115::*MFα*-*rBht*_(57–594)_ (N289Q)-*HIS*; row 16, GS115::*MFα*-*rBht*_(57–594)_ (N297Q)-*HIS*; row 17, GS115::*MFα*-*rBht*_(57–594)_ (N431Q)-*HIS;* row 18*,* GS115::*MFα*-*rBht*_(57–594)_ (N569Q)-*HIS;* row 20, GS115 (*His* ^+^) control. Error bars represent standard deviations from the means of three replicates. A single asterisk indicates a statistically significant increase was observed between two activity values (*p* < 0.05).

The high percentage of serine and threonine (29.1%) residues in the IDR (Figure 1B) provided the opportunity for phosphorylation as well as o-glycosylation and formed the basis for our deletion analysis strategy within the IDR. Most putative phosphorylation and o-glycosylation sites are “nested” between residues 32–54. Therefore, the first truncation involved the removal of amino acids 1–31 (rBHT_(32–594)_-HIS), prior to the nest of putative PTM sites. While removal of amino acids 1–53 (rBHT_(54–594)_-HIS) completely removed the putative PTM nest. The 1^st^ phosphorylation site found in the crystal structure (T56) was also removed rBHT_(57–594)_-HIS. The final 2 phosphorylation sites (T74 and T79) and α1 alpha helix were eliminated by removal of residues 1–81 (rBHT_(82–594)_-HIS). Finally, removal of residues 1 to 94 (rBHT_(95–594)_-HIS), and 1 to 102 (rBHT_(103–594)_-HIS) eliminated the β-turn (TT) and 3_10_ helix random coil (η1), respectively. Removal of the entire unique N-terminal residues 1–110 (rBHT_(111–594)_-HIS) was previously described (Dagher and Bruno-Bárcena, 2016). A schematic representation of the complete rBHT and rBHT-truncated variants is illustrated in Figure 2A.

**FIGURE 3:**
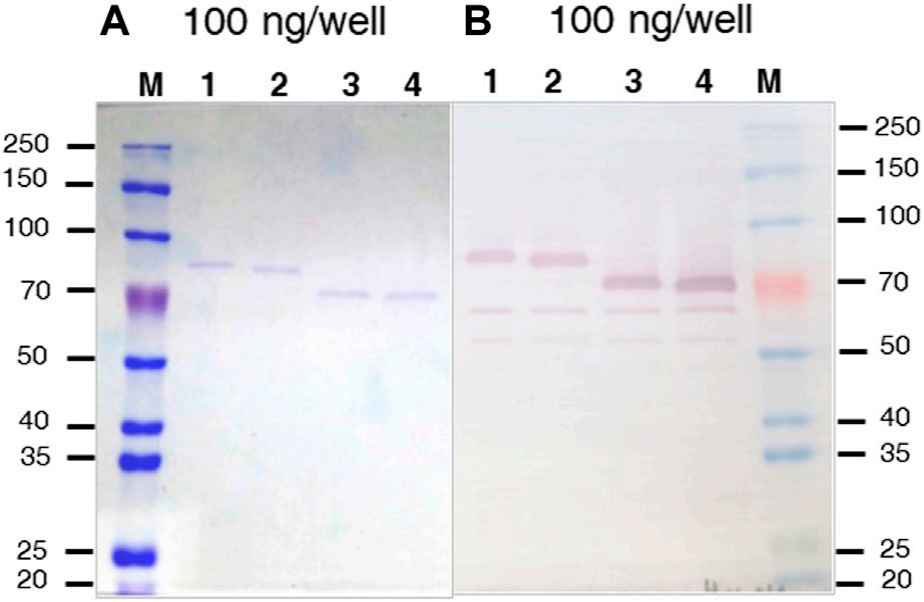
Coomassie stained SDS-PAGE and Western blot of rBHT-HIS deletion variants **(A)** SDS-PAGE (10%) stained with Coomassie blue. **(B)** Western blot exposed to anti-HIS antiserum of separated proteins. The Figures show protein cell free extracts (soluble secreted proteins) of *K. phaffii* GS115 expressing different recombinant BHT constructs generated by; lane 1, GS115::*MFα-rBht*_*(23–594)*_ *-HIS*; lane 2, GS115::*MFα-rBht*_*(32–594)*_ *-HIS*; lane 3, GS115::*αMF-rBht*_*(54–594)*_ *-HIS*, lane 4, GS115::*αMF-rBht*_*(57–594)*_ *-HIS.* Equal amounts (100 ng) were loaded in each lane to aid in the comparison. M indicates lane containing the molecular weight protein markers (Thomas Scientific, Swedesboro, NJ) and (kDa) shown to the left and right of the panels.

Methanol induced protein expression of each variant by *K. phaffii* GS115, for both membrane associated and soluble enzymes were evaluated as previously described (Dagher and Bruno-Bárcena, 2016). The presence of rBHT truncated protein variants in the broth (Figure 2B) was initially inspected by Coomassie stained SDS-PAGE (Figure 3A) followed by Western blot analysis (Figure 3B). Variants rBHT_(23–594)_-HIS, rBHT_(32–594)_-HIS, rBHT_(54–594)_-HIS and rBHT_(57–594)_-HIS were clearly detectable by Coomassie stain (Figure 3A) and Western blot (Figure 3B). While removal of additional fragments within the unique 110 region (rBHT_(82–594)_-HIS, rBHT_(95–594)_-HIS, rBHT_(103–594)_-HIS, and rBHT_(111–594)_-HIS) did not render detectable activity or protein using Coomassie stain or Western blot, indicating residues downstream of 57 (non-IDR regions) were important for processing secreted protein. In agreement with previous results, full length rBHT_(1–594)_-HIS variant was barely visible by Western blot (Dagher and Bruno-Bárcena, 2016). *K. phaffii* GS115 transformed with an empty pPIC9 vector was utilized as the negative control.

Most notable result was an approximately 30 kDa mobility shift on SDS-PAGE between rBHT_(32–594)_-HIS and rBHT_(54–594)_-HIS (Figure 3), possibly due to the deletion of the PTM nest containing predicted O-glycosylation (Figure 1B, GlycoEP) at positions (T34, S35, T39, T43, T50, T52) or phosphorylation (Figure 1B, DisPhos1.3) sites and surrounding acidic residues (Y37 (LTSN**Y**ETPS), T39 (SNYE**T**PSPT), S41 (YETP**S**PTAI), T43 (TPSP**T**AIPL), T50 (PLEP**T**PTAT), T52 (EPTP**T**ATGT)), known to retard proteins on SDS-PAGE (Lee et al., 2019).

An additional feature tested was the ability to drive secretion from predominantly membrane associated to soluble form. The secreted enzymatic activity associated with the membrane remained constant for rBHT_(23–594)_-HIS, rBHT_(32–594)_-HIS and rBHT_(54–594)_-HIS and rBHT_(57–594)_-HIS and no significant differences in ratio of soluble secreted versus membrane associated enzyme activity were observed for variants rBHT_(23–594)_-HIS, rBHT_(32–594)_-HIS and rBHT_(54–594)_-HIS. However, while rBHT_(57–594)_-HIS variant’s activity found associated with the membrane remained relatively constant, the ratio of secreted versus membrane associated enzyme activity increased between 25% and 40% (Table 4) when compared to variants rBHT_(23–594)_-HIS, rBHT_(32–594)_-HIS and rBHT_(54–594)_-HIS.

**TABLE 4:** Normalized enzyme activity comparison of (A) soluble *versus* (B) membrane bound secreted protein variants.

Enzyme Source		Mean values of secreted activity (mU/OD_600 nm_) ±SD^a^		(A/B) ratio secreted Soluble/Membrane bound
		(A) Soluble ± SD	(B) Membrane bound ± SD	
**1**	**GS115::*rBht***_**(1–594)**_ **-*HIS***	0.69 ± 0.10	10.63 ± 0.31	0.06
**2**	**GS115::*MFα*-*rBht***_**(1–594)**_ **-*HIS***	ND	ND	--
**3**	**GS115::*MFα*-*rBht***_**(23–594)**_ **-*HIS***	9.62 ± 0.20	24.04 ± 0.53	0.40
**4**	**GS115::*MFα-rBht***_**(32–594)**_ **-*HIS***	8.24 ± 0.12	25.84 ± 0.86	0.32
**5**	**GS115::*MFα*-*rBht***_**(54–594)**_ **-*HIS***	10.87 ± 0.30	30.23 ± 1.08	0.36
**6**	**GS115::*MFα*-*rBht***_**(57–594)**_ **-*HIS***	15.60 ± 0.66	29.52 ± 1.28	0.53
**7**	**GS115::*MFα*-*rBht***_**(82–594)**_ **-*HIS***	ND	ND	--
**8**	**GS115::*MFα*-*rBht***_**(95–594)**_ **-*HIS***	ND	ND	--
**9**	**GS115::*MFα*-*rBht***_**(103–594)**_ **-*HIS***	ND	ND	--
**10**	**GS115::*MFα*-*rBht***_**(111–594)**_ **-*HIS***	ND	ND	--
**11**	**GS115::*MFα*-*rBht***_**(23–594) (**_ **_N289Q_)-*HIS***	1.41 ± 0.02	5.77 ± 0.15	0.24
**12**	**GS115::*MFα*-*rBht***_**(23–594) (**_ **_N297Q_)-*HIS***	1.43 ± 0.00	4.88 ± 0.02	0.29
**13**	**GS115::*MFα*-*rBht***_**(23–594) (**_ **_N431Q_)-*HIS***	1.40 ± 0.06	5.48 ± 0.20	0.26
**14**	**GS115::*MFα*-*rBht***_**(23–594) (**_ **_N569Q_)-*HIS***	3.15 ± 0.07	4.90 ± 0.15	0.63
**15**	**GS115::*MFα*-*rBht***_**(57–594) (**_ **_N289Q_)-*HIS***	1.58 ± 0.06	8.95 ± 0.02	0.18
**16**	**GS115::*MFα*-*rBht***_**(57–594) (**_ **_N297Q_)-*HIS***	1.51 ± 0.04	9.00 ± 0.20	0.17
**17**	**GS115::*MFα*-*rBht***_**(57–594) (**_ **_N431Q_)-*HIS***	2.00 ± 0.06	7.69 ± 0.54	0.26
**18**	**GS115::*MFα*-*rBht***_**(57–594) (**_ **_N569Q_)-*HIS***	1.96 ± 0.25	9.13 ± 0.54	0.21
**19**	**GS115 control**	ND	ND	--

a The value of cell density (OD_600nm_) reached by the recombinant strains (Enzyme Source) after methanol induction was used to normalize the secreted soluble and membrane-bound activities. The maximum cell densities obtained were between 60 and 75 OD_600nm_. The results are mean values for three measurements of enzyme activity and standard deviation (SD). “ND” indicates enzyme activity was not detected.

Under our experimental conditions, the results showed undetectable amounts of soluble, or membrane associated active protein when residues downstream of the IDR were removed (Table 4). To further evaluate whether bioactive rBHT_(82–594)_-HIS, rBHT_(95–594)_-HIS and rBHT_(103–594)_-HIS variants, were produced and secreted in low amounts, inductions of the corresponding cell lines were performed, and culture broth was concentrated 100-fold. However, neither soluble nor cell-associated rBHT from those variants had any enzymatic activity. This may be caused by ineffective secretion or destruction of the protein molecules that were not secreted.

The combined results strengthen a new finding that the BHT IDR by itself is not directly responsible for enzymatic activity or membrane interactions (Table 4).

### Kinetic parameters of secreted rBHT variants

Since the initial analysis of truncated variants revealed greater rBHT_(57–594)_-HIS titers (Table 4), we investigated if the structural organization exhibits kinetic biases or, more specifically, whether the IDR more broadly affects protein kinetics.

Active soluble secreted rBHT variants were functionally assessed by conventional kinetic measurements after being purified to homogeneity utilizing a C-terminal 6xHistidine epitope and Nickel affinity chromatography. Examination of the isolated rBHT variants by SDS-PAGE separation under reducing conditions showed the proteins were essentially homogenous (data not shown).

**FIGURE 4:**
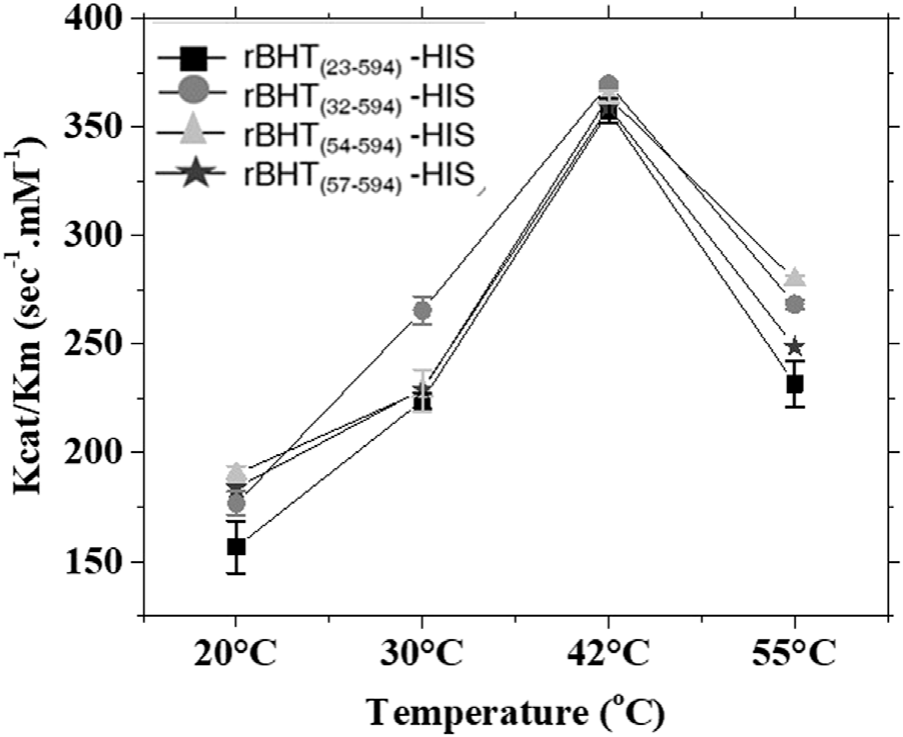
Enzyme kinetic parameters for rBHT variants tested at 20°C, 30°C, 42°C and 55°C. Enzyme assays depicted as kcat/km versus temperature were carried out in the presence of 0.3 µg rBHT_(23–594)_-HIS, rBHT_(32–594)_-HIS, rBHT_(54–594)_-HIS and rBHT_(57–594)_-HIS over a range of ONP-Glc substrate concentrations (0.08–10.4 mM). Assays were described under “Methods.” *Km* and *kcat* were calculated from initial velocities of ONP-Glc cleavage using the Hill equation with a Hill coefficient of 1. The values are the average of three independent measurements ± Standard Deviation (SD).

The kinetic parameters of each active secreted soluble variant, rBHT_(23–594)_-HIS, rBHT_(32–594)_-HIS, rBHT_(54–594)_-HIS, and rBHT_(57–594)_-HIS were investigated. To obtain a full kinetic picture, an important parameter evaluated was the impact of temperature on enzymatic activity. Therefore, assays were performed at the optimum temperature for rBHT_(23–594)_-HIS of 42°C (Dagher and Bruno-Bárcena, 2016), below 42°C (20°C and 30°C) and above 42°C (55°C) (Figure 4). The results of the respective *kcat/km* for all four truncated enzyme variants indicate a temperature optimum of 42°C (Figure 4). At each temperature tested all enzyme-truncated variants maintain a similar affinity for the substrate ONP-Glc (Km) and turnover activity (kcat) therefore confirming that truncations within the IDR do not play a role in the catalytic integrity of rBHT.

### Are N -glycans essential for secretion?

Proteome-wide investigations have revealed relationships between IDRs and several PTMs, including acetylation, methylation, and glycosylation (Dunker et al., 2013). Extensive *in silico* analysis revealed there are only 4 predicted N-linked glycosylation sites out of a possible 19, and none were discovered in the 110-residue N-terminus that contains the IDR (Figure 1). Investigations have indicated that glycosites are found mostly in structured sections, some distance from the disordered stretches, which is consistent with our findings (Singh et al., 2018; Goutham et al., 2020). All four N-glycosylation sites are in highly conserved glycosylation consensus sites (Asn-X-Ser/Thr X≠Pro) and nearby residues in the crystal structures of rBHT_(23–594)_-HIS (BHT, PDB: 7L74 and HsBglA, PMB: 6M4E), indicating a high likelihood of functionally relevant glycosylation at those positions **N**289LTY, **N**297STS, **N**431QSD, and **N**569QSD.

We examined each of the four N-glycosylation sites of BHT to determine if they had any functional importance. By employing *rBht*_(23–594)_-*HIS* as a template for site-directed mutagenesis, the asparagine residues (N289, N297, N431 and N569) were separately changed to glutamine residues to abrogate glycosylation, as explained in Materials and Methods. The results showed significant reductions of secreted soluble enzyme activities up to (90%, 95% and 97%) from three variants GS115::*MFα-rBht*_*(23–594) (N431Q)*_ *-HIS,* GS115::*MFα-rBht*_*(23–594) (N289Q)*_ *-HIS* and GS115::*MFα-rBht*_*(23–594) (N297Q)*_ *-HIS* when compared to non-mutated variant GS115::*MFα-rBht*_*(23–594)*_ *-HIS* activity, respectively. When compared to the GS115::*MFα-rBht*_*(23–594)*_ *-HIS* version, the GS115::*MFα-rBht*_*(23–594) (N569Q)*_ *-HIS* variant demonstrated a less pronounced activity loss of 67% and an enhanced ratio of secreted to cell membrane associated activity from 0.40 to 0.63 (Table 4). When compared to the parent strain GS115::*MFα-rBht*_*(23–594)*_ *-HIS*, cell membrane associated activity for the strains GS115::*MFα-rBht*_*(23–594) (N289Q)*_ *-HIS*, GS115::*MFα-rBht*_*(23–594) (N297Q)*_ *-HIS,* GS115::*MFα-rBht*_*(23–594) (N431Q)*_ *-HIS,* and GS115::*MFα-rBht*_*(23–594) (N569Q)*_ *-HIS*, decreased by up to 81%, 95%, 84%, and 75%, respectively (Table 4). When the complete IDR is removed (GS115::*MFα-rBht*_*(57–594) (N569Q)*_ *-HIS*), similar outcomes are shown. Notably, membrane-bound related activity was markedly reduced but not eliminated, indicating that glycosylation affects cell membrane localization and secretion.

We purified each N-glycosylation mutant by Ni-chromatography and confirmed that the variants had similar specific activities as the non-mutated forms (Table 4). Therefore, altering the N-glycosylation site interfered with secretion but did not alter the activity of the variants.

### Interface analysis

**FIGURE 5:**
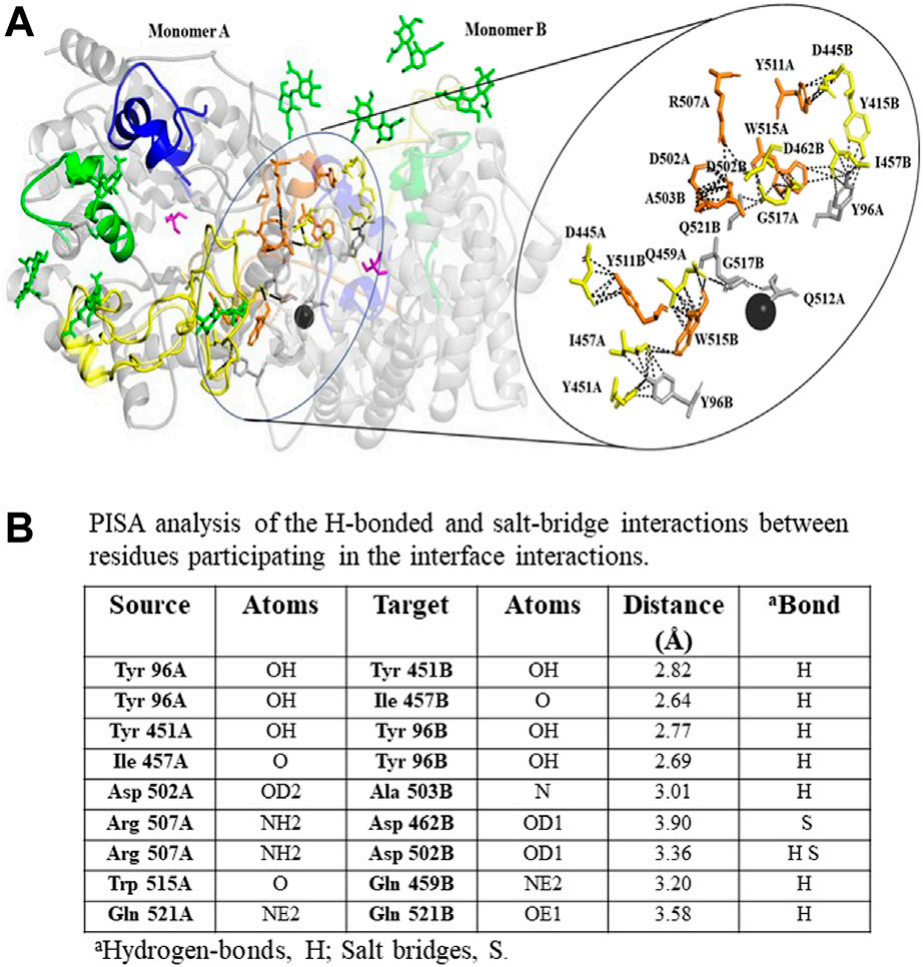
Structural organization of rBht_(23–594)_-HIS dimer interface. **(A)** A ribbon representation of rBht_(23–594)_-HIS dimer highlighting loops (A; blue, B; green, C; yellow, D; orange) surrounding the active site is shown on the left. The inset on the right shows the dimer interface in greater detail with predicted salt bridges and H-bonds as black dashed lines. **(B)** Types of interface bonds and distances. The TRIS molecules occupying the −1 subsite is represented as a magenta stick. NAG are shown as green sticks. Ca^2+^ ion is represented as a black sphere. The figure was produced using PyMOL (https://pymol.org/2/) (Schrodinger, 2022). PDBePISA (https://www.ebi.ac.uk/pdbe/pisa/picite.html) was used to predict interface bonds and distances (Krissinel and Henrick, 2007).

rBHT was crystallized in the C2 space group with two molecules per asymmetric unit, suggesting a possible dimer (Table 3). The same dimer is found in 6M4E, though in 6M4E a crystallographic axis of 2-fold symmetry runs through the dimer leading to only one molecule per asymmetric unit (Uehara et al., 2020). To distinguish between significant crystal interface interactions and artifacts of crystal packing (Krissinel, 2010) we used the program PISA (Protein Interfaces, Surfaces, and Assemblies) which calculates interface stability and entropy of dissociation to identify stable chemical contacts (Krissinel and Henrick, 2007). Analysis of PDB: 7L74 revealed a buried surface area of 7,803.5Å^2^ between molecules A and B. The interface between molecules A and B has the largest negative Δ^i^G (−21.3 kcal/mol) so it is predicted to be the strongest and with dissociation energy ΔG^diss^ (23.8 kcal/mol) and corresponded with the experimental results shown below. Residues in Loops C (yellow) and D (orange) contribute to most of the protein-protein interaction formed between the monomers (Figure 5). The residues involved in this interaction include hydrogen bond formation and salt bridges as shown in Figure 5B and depicted as yellow, orange, and gray sticks (Figure 5A).

### Influence of N-terminal deletions on rBHT dimerization

**FIGURE 6:**
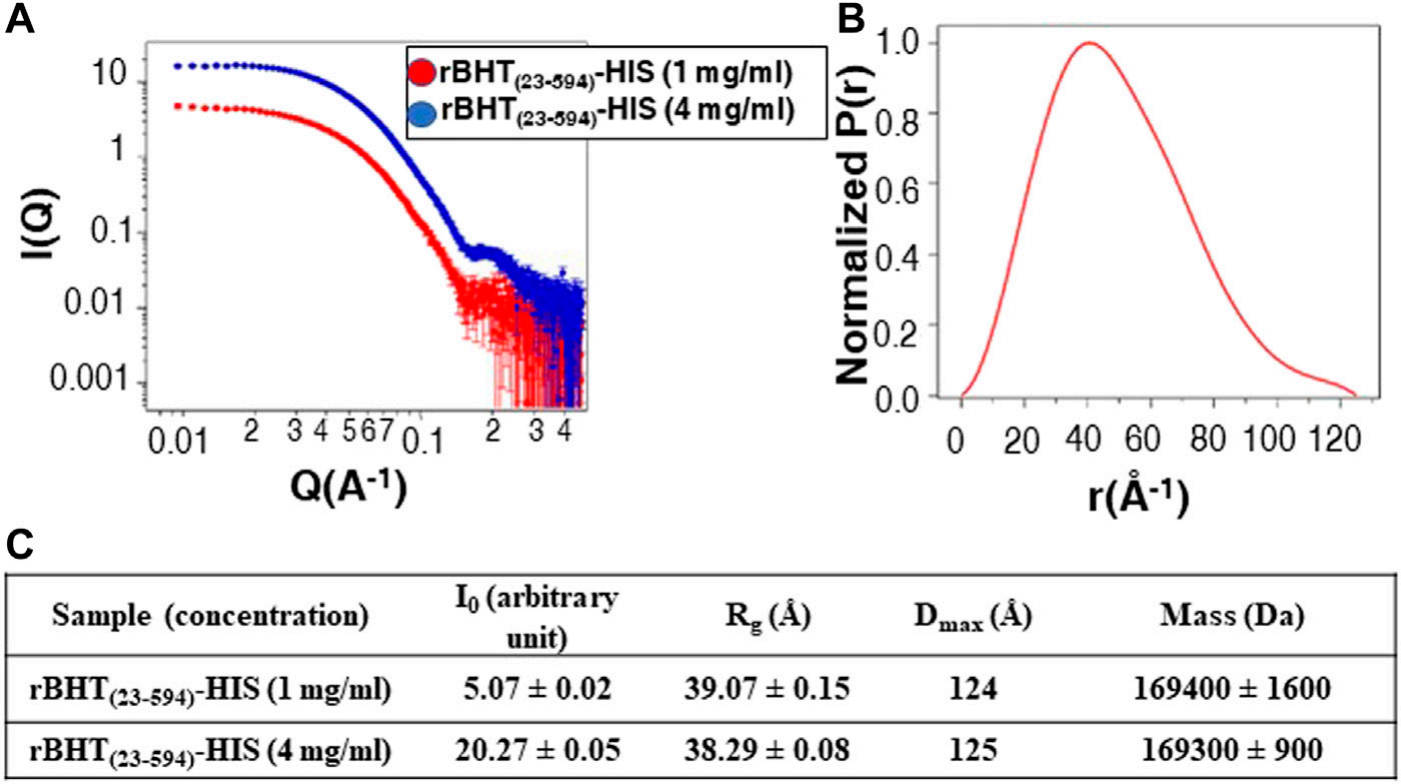
SAXS data for rBHT_(23–594)_-HIS at 1 mg/mL and 4 mg/mL. **(A)** SAXS data are shown on a log–log plot (left). *I(Q)* is in arbitrary units. **(B)** P(r) curve calculated from the SAXS data are normalized to a maximum height of 1.0. **(C)** Solution scattering parameters zero-angle intensity *I*_*0*_, radius of gyration *R*_*g*_, and maximum dimension *D*_*max*_ and SAXS-calculated molecular weight for rBHT_(23–594)_-HIS at 1 mg/mL and 4 mg/mL.

To gain further insights into the oligomerization properties of BHT, we performed small X-ray scattering (SAXS) analysis (Figure 6A). Guinier and *P(r)* analysis was performed using PRIMUS and GNOM, respectively (Svergun, 1992; Konarev et al., 2003). *D*_*max*_ values were manually chosen in GNOM to optimize the P(r) calculation (Figure 6B). These *D*_*max*_ values are approximate to ∼±2–3 Å. Molecular mass were calculated using the method described by Rambo and Tainer (Rambo and Tainer, 2013). The data are presented in Figure 6C. The molecular mass determined from SAXS (∼169 KDa) confirmed that rBHT forms a dimer in solution (Figure 6C). The *R*_*g*_ and *D*_*max*_ of the dimer in solution are 39 Å and 124 Å, respectively. As stated above, the X-ray crystallographic structures (PDB: 7L74, 6M4E, 6M4F and 6M55) also suggest that rBHT forms a dimer. The *R*_*g*_ and *D*_*max*_ of the PDB: 7L74 crystallographic dimer (molecule A and molecule B) calculated using Crysol are 34 Å and 110 Å, respectively (Svergun et al., 1995). These values are in agreement with the experimental SAXS data. The disordered N-terminus led to a more expanded dimer in solution, and we conclude that rBHT_(23–594)_-HIS likely functions as a dimer. The SAXS experimental data have been deposited in the Small-Angle Scattering Biological Data Bank (SASBDB) (https://www.sasbdb.org/) under accession codes SASDN57 for rBHT_(23–594)_-HIS, 1 mg/mL and SASDN67 for rBHT_(23–594)_-HIS, 4 mg/mL.

The dimer conformation was further validated in solution by size exclusion chromatography (SEC). The variants rBHT_(23–594)_-HIS, rBHT_(32–594)_-HIS, rBHT_(54–594)_-HIS, and rBHT_(57–594)_-HIS subjected to SEC showed the rBHT variants eluted as a single peak having a retention time of 12.5 min with calculated M_w_ 150 kDa demonstrating rBHT_(23–594)_-HIS, rBHT_(32–594)_-HIS, rBHT_(54–594)_-HIS, and rBHT_(57–594)_-HIS all exist as dimers in solution (Data not shown). The confirmation of dimer formation of rBHT_(23–594)_-HIS, rBHT_(32–594)_-HIS, rBHT_(54–594)_-HIS, and rBHT_(57–594)_-HIS, suggested that the unstructured regions spanning residues 23–56 are not involved in dimerization. Based on these analysis the IDR of rBHT does not appear to be essential for dimer formation or secretion of active enzyme.

## Discussion

Here, we investigated whether the presence of the distinctive N-terminal intrinsically disordered region (IDR) and/or putative posttranslational modifications in the GH1 C-terminal domain affect the amount of secreted active BHT. These results confirm that the rBHT IDR is not essential for activity or drive protein-membrane interactions.

Because the crystal structure did not provide any observable electron density at the N-terminal residues 23 to 53, IUPRED2A was used to predict the C-terminal border of the disordered region at amino acid 56. Deletion variants were generated based on the expected disordered portions until all 56 N-terminal residues were removed. Although native BHT is a membrane-associated protein, all rBHT variations partitioned between soluble secreted and cell membrane associated forms. Furthermore, soluble secreted enzyme variants that were shorted up to residue 56 displayed comparable catalytic properties. Although additional N-terminal deletions variants were not detected, it is possible that their removal affected rBHT’s stability or secretory pathway. Disordered regions can be discriminated from ordered ones based on the amino acid sequence (Garner et al., 1998; Darling and Uversky, 2018) and in most cases, disordered proteins are less evolutionarily conserved but rather their disordered structure has been maintained (Brown et al., 2011). Previously reviewed data indicated that low sequence complexity, high net charge, and low concentration of hydrophobic residues are a hallmark of disordered protein regions employed for interactions with lipid bilayers (Theillet et al., 2013; Cornish et al., 2020). However, a significant elemental preference for disorder-promoting residues reported in classical IDRs is called into doubt by the high proportion of hydrophobic residues in the BHT IDR (35.6%), placing it within the category of molecular recognition features (MoRFs) (Theillet et al., 2013; Yan et al., 2015).

Beyond the secretion signal sequences chosen, several other factors also govern protein secretion. For instance, the release of heterologous proteins depends on N-glycosylation, a post-translational modification involved in protein folding in the ER (Skropeta, 2009). It was therefore crucial to conduct additional research on the relationship between rBHT N-glycosylation and enzymatic properties to assess the stability, activity, and even secretion of the enzyme. However, not all polypeptides with predicted N-glycosylated sequons are glycosylated *in vivo*. Finding the locations of the N-linked glycosylation sites in the C-terminal GH1 domain was made easier by solving the crystal structure of rBHT_(23–594)_-HIS. No N-glycosylation sites were predicted in the IDR region, even though algorithms were useful at predicting O-glycosylation sites within the IDR (Figure 1). In this study, *in vivo* analyses were primarily used to evaluate the impact of eliminating a putative glycosylation site on expression, secretion, and activity. Although it appears that glycosylation is not necessary for enzymatic activity, the significant decrease in overall protein secretion observed for each of the four variants suggested that glycosylation may provide protection by increasing protein stability, shielding exposed hydrophobic surfaces, reducing proteolysis, and even increasing solubility.

When associated to the membrane, BHT must be conformationally flexible, whereas when unconnected to the membrane, it must be stable. Given that rBHT homodimer activity and stability when expressed by *K. phaffii* GS115 are independent of the N-terminal 56 amino acids, it is possible that elements (PTMs) in addition to unique amino acids within the catalytic domain may serve as a handle for specific catalytic advantages in preserving the active enzyme.

## Data Availability

The datasets presented in this study can be found in online repositories. The names of the repository/repositories and accession number(s) can be found below: http://www.wwpdb.org/, 7L74 https://www.sasbdb.org/, Small-Angle Scattering Biological Data Bank (SASBDB) under accession codes SASDN57 and SASDN67 (https://www.sasbdb.org/).

## Abbreviations

BHT, native ß–hexosyltransferase; rBht, optimized coding sequence; rBHT, expressed recombinantly; Glc, glucose; GlcNAc, N-acetylglucosamine; Man, mannose; Asn, asparagine; Ser, serine; Thr, threonine; MFα, mating factor α; GH1, glycosyl hydrolase 1; PTM, post-translation modifications; SD, Standard Deviation; ONP-Glc, oNP-β-D-glucopyranoside.
